# Insights into epigenetic patterns in mammalian early embryos

**DOI:** 10.1007/s13238-020-00757-z

**Published:** 2020-07-15

**Authors:** Ruimin Xu, Chong Li, Xiaoyu Liu, Shaorong Gao

**Affiliations:** 1 Clinical and Translational Research Center of Shanghai First Maternity and Infant Hospital, Shanghai Key Laboratory of Signaling and Disease Research, Frontier Science Center for Stem Cell Research, School of Life Sciences and Technology, Tongji University, Shanghai, 200092 China; 2 Institute for Regenerative Medicine, Shanghai East Hospital, Shanghai Key Laboratory of Signaling and Disease Research, Frontier Science Center for Stem Cell Research, School of Life Sciences and Technology, Tongji University, Shanghai, 200092 China; 3 Tsingtao Advanced Research Institute, Tongji University, Qingdao, 266071 China

**Keywords:** epigenetic reprogramming, DNA methylation, histone modifications, early embryo development

## Abstract

Mammalian fertilization begins with the fusion of two specialized gametes, followed by major epigenetic remodeling leading to the formation of a totipotent embryo. During the development of the pre-implantation embryo, precise reprogramming progress is a prerequisite for avoiding developmental defects or embryonic lethality, but the underlying molecular mechanisms remain elusive. For the past few years, unprecedented breakthroughs have been made in mapping the regulatory network of dynamic epigenomes during mammalian early embryo development, taking advantage of multiple advances and innovations in low-input genome-wide chromatin analysis technologies. The aim of this review is to highlight the most recent progress in understanding the mechanisms of epigenetic remodeling during early embryogenesis in mammals, including DNA methylation, histone modifications, chromatin accessibility and 3D chromatin organization.

## Introduction

Fertilization is regarded as one of the greatest feats of nature, beginning with a sperm combining with an oocyte, during which these two terminally differentiated germ cells are converted into a totipotent zygote (Rivera and Ross, 2013; Canovas and Ross, 2016; Xu and Xie, 2018). Dramatic changes occur to ensure that a series of pivotal biological events proceed, including oocyte activation(Amdani et al., 2015; Yeste et al., 2017; Bonte et al., 2018) and maternal-to-zygotic transition (MZT) coordinated with zygotic gene activation (ZGA) (Minami et al., 2007; Tadros and Lipshitz, 2009; Jukam et al., 2017; Eckersley-Maslin et al., 2018; Schulz and Harrison, 2019), followed by the first cell-fate decision and lineage-specific differentiation (Mihajlovic and Bruce, 2017; Zhang et al., 2018; Yao et al., 2019). A precise regulatory network must function appropriately to support such a major transition in a brief period. Epigenetic information plays a major role in the maintenance of cell identity and the control of gene expression. Epigenetic modifications in terminally differentiated gametes, including DNA methylation (Guo et al., 2014b; Iurlaro et al., 2017; Zhu et al., 2018; Zeng and Chen, 2019), histone modifications (Dahl et al., 2016; Liu et al., 2016b; Zhang et al., 2016; Inoue et al., 2017a; Wang et al., 2018a), modifications affecting chromatin accessibility (Wu et al., 2016; Jachowicz et al., 2017; Gao et al., 2018a) and 3D chromatin organization (Du et al., 2017; Ke et al., 2017; Kragesteen et al., 2018), can be reset to a basal state after fertilization to achieve totipotency and support development into a new individual. Unexpected changes in the external environment may lead to irreversible damage to proper growth by altered epigenetic patterns that may interfere with gene expression (Legault et al., 2018; Risal et al., 2019; Yu et al., 2019).

Recently, methodological advances such as low-input chromatin analysis technologies (Xu and Xie, 2018) have provided approaches to overcome the inaccessibility of early-stage embryos and elucidate the epigenetic remodeling mechanism at the whole-genome level. In this review, we will discuss the dynamic processes of epigenetic re-establishment during mouse and human early embryonic development, focusing on how these epigenetic mechanisms may promote the acquisition of totipotency and the differences between mice and humans in these reprogramming events.

## DNA METHYLATION

### Large-scale asymmetric demethylation upon mouse fertilization

DNA methylation (5mC) is an inheritable type of epigenetic mark that provides molecular memory to preserve the transcriptional order during mammalian early embryo development (Li and Zhang, 2014; Messerschmidt et al., 2014; Okae et al., 2014a; Zeng and Chen, 2019). The methylation of cytosine residues is catalyzed by the de novo DNA methyltransferases (DNMT3A/B) and maintenance DNA methyltransferase (DNMT1), while TET family enzymes act in a multistep process to achieve DNA demethylation (Okano et al., 1999; Kohli and Zhang, 2013; Wang et al., 2014; Wu and Zhang, 2014, 2017; Verma et al., 2018). Before the first cleavage, both the maternal and paternal genomes undergo widespread active and passive demethylation, except in imprinting control regions (ICRs) and some retrotransposons (Smith et al., 2012; Guo et al., 2014a; Messerschmidt et al., 2014; Shen et al., 2014; SanMiguel and Bartolomei, 2018). The paternal genome is more rapidly and actively demethylated, along with the exchange of protamines for maternal histones, including HIRA-mediated H3.3 deposition (Loppin et al., 2005; Skene and Henikoff, 2013; Inoue and Zhang, 2014).

Immunofluorescence data show that the paternal genome loses the 5mC signal before the first DNA replication of the zygote at pronuclear stage 3 (PN3) (Oswald et al., 2000). Further investigation suggests that paternal 5mC is converted to 5hmC by TET3 and then removed via replication-coupled passive dilution (Gu et al., 2011; Inoue and Zhang, 2011). However, another study on mouse zygotes shows that the early loss of paternal 5mC is unaffected when 5hmC formation is abrogated by small-molecule inhibition of TET activity (Amouroux et al., 2016). TET3 seems to be unrequired for loss of 5mC in the early zygote. Instead, TET3 targets new 5mC generated by DNMT3A and DNMT1, which indicates that accumulation of paternal 5hmC is resulted from *de novo* DNA methylation rather than the TET3-driven hydroxylation of paternal 5mC (Amouroux et al., 2016). This provides another possible mechanism determining paternal DNA methylation dynamics. The maternal genome is more resistant to this initial wave of demethylation, di-methylated histone H3 lysine 9 (H3K9me2) is reported to promote CG methylation maintenance in maternal genome by recruiting the maternal factor PGC7 (also known as STELLA, DPPA3 (Nakamura et al., 2007; Nakamura et al., 2012; Han et al., 2018; Zeng et al., 2019). However, the appropriateness of this conclusion is queried by a recent study which shows a global increase in 5mC level induced by uncontrolled *de novo* methylation by DNMT1 in STELLA-deficient zygotes (Li et al., 2018b). Besides, another study shows H3K9me2 enrichment is reduced by oocyte specific deletion of G9a (an H3K9me2 methyltransferase), but the CG methylation is minimally affected (Au Yeung et al., 2019). Thus, the role of H3K9me2 and *Stella* in DNA methylation protection of the maternal genome are worthy of further verification. In addition, the maternal genome is more prone to passive demethylation during sequential cleavages, which is DNA replication dependent, giving rise to epigenetic asymmetry in the early embryo (Stewart et al., 2016; Guo et al., 2017; Xu and Xie, 2018).

Within the developing embryos, the initial global hypomethylation level maintains naïve pluripotency and guarantees accurate future differentiation regulation (Nichols and Smith, 2009; Theunissen et al., 2016; Peng et al., 2019). DNA methylation is re-established in lineage-specific regions beginning in the blastocyst stage (Zhang et al., 2018). The exit from pluripotency and entry into lineage-specific differentiation have been proven to be associated with genome-wide *de novo* DNA methylation, during which the co-expression of the DNMT3 and TET enzymes promotes coherent genome-wide oscillations of CpG-density-dependent DNA methylation (Smith et al., 2017; Rulands et al., 2018). These findings provide insights into the emergence of epigenetic heterogeneity during early embryo development, indicating that dynamic changes in DNA methylation might influence early cell fate decisions (Rulands et al., 2018).

Aberrant reprogramming of the DNA methylome may lead to developmental defects and embryonic arrest. In somatic cell nuclear transfer (SCNT) embryos, the efficiency of embryonic development is much lower than that in normally fertilized embryos, owing to the multiple epigenetic barriers that impede SCNT-mediated reprogramming, including abnormally higher levels of DNA methylation in cloned embryos (Dean et al., 2001; Yang et al., 2007; Peat and Reik, 2012; Teperek and Miyamoto, 2013). By comparing the DNA methylome of SCNT embryos and fertilized embryos, differentially methylated regions (DMRs) are identified (Gao et al., 2018b). Unexpected re-methylation is found in cloned embryos, which possesses higher methylation level than the former stage, termed as re-methylated DMRs (rDMRs). rDMRs are enriched at promoters, SINE and long terminal repeat (LTR). Re-methylation-affected downregulated genes at 2-cell stage of SCNT embryos are highly enriched of totipotent- and developmental-related genes (Gao et al., 2018b). This finding is compatible with the perspective that an aberrant DNA methylome at the ZGA stage may lead to developmental defects and female infertility (Wang and Dey, 2006). Moreover, the totipotency marker Mouse endogenous retrovirus type L (MERVL), one of the endogenous retroviruses (ERVs) specifically expressed at the 2-cell stage (Svoboda et al., 2004; Ribet et al., 2008; Eckersley-Maslin et al., 2016; Huang et al., 2017), exhibits a dramatically higher remnant methylation state in SCNT embryos than in fertilized embryos, and its transcription activity was evidently repressed (Gao et al., 2018b). These observations collectively suggest that the impact of methylation memory from gametes or donor cells should be considered when investigating the epigenetic reprogramming of offspring.

### Generally similar reprogramming patterns but with different details in human pre-implantation embryos to those in mouse

Abundant studies on the reprogramming of the DNA methylome have paved the way for deciphering the mechanism of DNA methylation reprogramming in early human embryos (Fulka et al., 2004; Lister et al., 2009; Molaro et al., 2011; Smith and Meissner, 2013; Guo et al., 2014b). However, compared to the commonly utilized mammalian animal models, human embryos show a much more complex genetic background, which may present barriers to their analysis. Remarkable work has been accomplished on the genome-wide profiling of DNA methylation in human pre-implantation embryos, yet multiple issues remain to be addressed. In contrast to previous observations in mice, which show genome-wide demethylation occurs mainly at the 1-cell stage, the initial rapid DNA demethylation occurs from fertilization to 2-cell stage in human embryos and keep stable until the morula stage, then followed by a second reduction from morula stage to blastocyst stage (Guo et al., 2014b). A set of transient, maternal DMRs are found both in mouse and human early embryos, but more DMRs are resolving to hypermethylation in human and most of these short-lived DMRs are not equivalently regulated in mouse. Also, the repetitive element regulation is more diverse in human than that in mouse (Smith et al., 2014). Notably, the human paternal genome undergoes demethylation at a much faster rate than the maternal genome, which is similar to the situation in early mouse embryos (Fulka et al., 2004; Okae et al., 2014a; Smith et al., 2014; Zhu et al., 2018). The paternal genome shows rather lower DNA methylation level than the maternal one by the end of the zygotic stage (Fulka et al., 2004; Okae et al., 2014a; Smith et al., 2014). Moreover, H3K4me3-marked active genes in human ESCs are essentially devoid of DNA methylation in both mature gametes and human pre-implantation embryos (Guo et al., 2014b). Taking advantage of single-cell post-bisulfite adapter tagging (PBAT) DNA methylome sequencing analysis at the single-cell level and single-base resolution, issues such as the inclusion of aneuploid embryos in samples and the heterogeneity of DNA methylation among individuals can be addressed (Zhu et al., 2018). The global DNA methylation pattern displays a dynamic balance between dramatic demethylation and focused intensive de novo methylation. The major demethylation occurs in a stepwise and wave-like manner, while focused de novo methylation prevails at the 8-cell stage. Furthermore, the maternal genome is maintained in a more hypermethylated state at a wide variety of genomic loci during pre-implantation. In summary, these findings paved the way for revealing the mechanisms of the regulatory network of DNA methylation during early human embryogenesis. It remains to be determined whether the distinct features of asymmetric DNA methylomes may affect the transcriptome or play a role in cell fate decisions.

## HISTONE MODIFICATIONS

Histone modifications are key regulatory events throughout pre-implantation embryogenesis that influence the interactions of transcriptional regulators with chromatin (Stewart et al., 2015; Skvortsova et al., 2018; Wu et al., 2018; Xu and Xie, 2018). However, previous studies on histone modification reprogramming during mammalian early embryo development are restricted to observations based on immunofluorescence staining, which exhibits a relatively low resolution for the analysis of underlying mechanisms.

Taking advantage of the newly launched low-input ChIP-seq and CUT&RUN methods, a high-resolution map for investigating the reprogramming of histone modifications during mammalian pre-implantation embryos was finally profiled. Here, we review recent progress in understanding the molecular mechanisms of histone modification reprogramming in early embryogenesis generated by these newly developed methods.

### H3K4me3

#### A unique pattern in mouse oocytes and major resetting of H3K4me3 after fertilization

**Figure 1:**
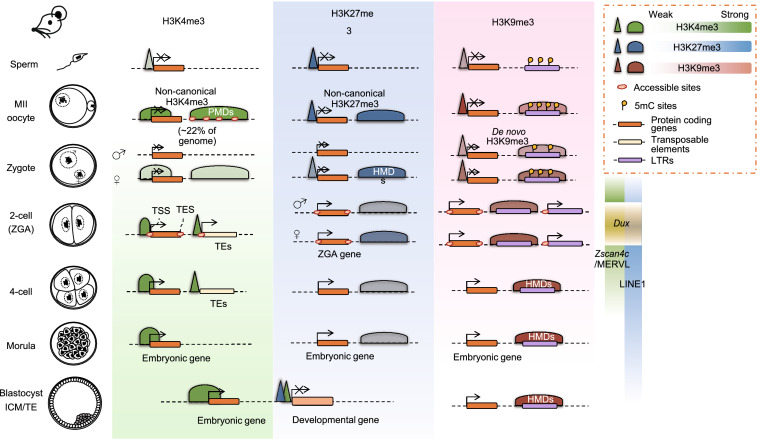
**Epigenome reprogramming of histone modifications and chromatin accessibility during early mouse embryo development.** H3K4me3: After fertilization, H3K4me3 in the paternal genome is rapidly depleted but re-established during major ZGA. By contrast, noncanonical H3K4me3 with broad domains in both promoters and distal regions is found in MII oocytes and is replaced by canonical H3K4me3 at the late 2-cell stage (ZGA). Broad ncH3K4me3 domains are correlated with partial DNA methylation domains (PMDs). Broad promoter H3K4me3 domains are much more abundant in early embryos than in MII oocytes or derived cell lines and are associated with high levels of gene expression. During ZGA, chromatin accessibility is observed at both the TSS and TES sites of active genes. Transposable elements (TEs) are also accessible and are enriched in distal H3K4me3. Developmental genes are primed to be active until the blastocyst stage, marked by bivalent H3K4me3/H3K27me3. H3K27me3: During mouse early embryo development, H3K27me3 in promoter regions exhibits a widespread loss at the 2-cell stage, a caused by the global erasure of H3K27me3 in the paternal genome and the selective depletion of promoter H3K27me3 in the maternal genome. H3K27me3 and DNA methylation are negatively correlated with H3K4me3. H3K27me3 also appears in non-promoter regions in a highly pervasive and promiscuous manner. H3K9me3: H3K9me3 peaks mainly fall in LTRs in early embryos. The number of H3K9me3-marked LTRs gradually increases and remains high during pre-implantation development. Most of the parental H3K9me3 regions are established *de novo* upon fertilization. Promoter H3K9me3 marks are erased upon fertilization and are reestablished postimplantation. Most LTR-enriched H3K9me3 domains are progressively established after the 4-cell stage and are responsible for LTR silencing. During the early cleavage stage, H3K9me3 domains overlap with H3K27me3-marked facultative heterochromatin. Maternal-specific H3K9me3 regions are much more abundant than paternal-specific regions during early embryogenesis, but this divergence gradually diminishes. Transposable elements: Mouse endogenous retrovirus type L (MERVL), a member of the ERV3 family member, is expressed in both 2-cell-like ESCs and cleavage-stage embryos, where it drives the expression of many transcripts specific to ZGA and totipotency. Upon fertilization, LINE1 is actively transcribed, with the increase in LINE1 RNA reaching its highest level at the 2-cell stage. LINE1 is essential for *Dux* silencing, the synthesis of rRNA, exit from the 2-cell stage and chromatin remodeling over accessible regions during pre-implantation embryo development. Chromatin accessibility: Open chromatin exists around both the promoters and transcription end sites (TES) of actively transcribed genes at the 2-cell stage. The transient and active transcription of transposable elements is probably associated with increased chromatin accessibility at the 2-cell stage of early embryos

Histone modifications play critical roles in the spatiotemporal regulation of gene expression in mammals (Bannister and Kouzarides, 2011; Sadakierska-Chudy and Filip, 2015; Huang et al., 2019; Xia et al., 2019). Recently, low-input ChIP-seq methods were developed and utilized to profile the genome-wide landscape of histone modifications during ZGA and the first cell fate decision in mouse early embryos (Dahl et al., 2016; Liu et al., 2016b; Zhang et al., 2016) (Fig. 1). After fertilization, H3K4me3 (a mark of active promoters) in the paternal genome is rapidly depleted, but this mark is re-established during major ZGA. By contrast, the noncanonical form of H3K4me3 (ncH3K4me3), covering broad domains in both promoters and distal regions, is found in the maternal genome (making up ~ 22% of the genome) (Dahl et al., 2016; Zhang et al., 2016). ncH3K4me3 is readily established in mature oocytes and is not replaced by canonical H3K4me3 until the major ZGA stage. The establishment and removal of distal ncH3K4me3 is a unique characteristic of oocyte and early embryo genomes. However, the mechanism and function of ncH3K4me3 dynamics still need to be further investigated. DNA methylation in the maternal genome is anti-correlated with ncH3K4me3, as ∼ 18% versus ∼ 57% CpG methylation is observed in oocytes with or without ncH3K4me3 (Wang et al., 2014; Dahl et al., 2016). Active removal of broad H3K4me3 domains by the lysine demethylases KDM5A and KDM5B is essential for normal ZGA and early embryogenesis(Dahl et al., 2016), while *Kdm5b* overexpression results in transcriptome reactivation in mature oocytes (Zhang et al., 2016), suggesting that ncH3K4me3 may be responsible for the genome-wide silencing state. Transposable elements (TEs), including B1/B2/B4 and ERVL, show high overlap with ncH3K4me3 in distal regions, indicating that ncH3K4me3 in distal regions can be correlated with the activities of repeats (Zhang et al., 2016). ncH3K4me3 is reprogrammed upon ZGA at the late 2-cell stage, which requires both zygotic transcription and active demethylation, rather than passive dilution (Zhang et al., 2016). Maternal factors within the oocyte cytoplasm must direct the rapid change of the H3K4me3 landscape. Identifying key maternal factors may provide more insight into the mechanism of H3K4me3 remodeling. On the other hand, H3.3 is cooperated to the genome after fertilization and is essential for embryo development (Lin et al., 2013; Lin et al., 2014; Wen et al., 2014). The turnover of H3.3 is associated with histone modification changes such as H3K27me3 and H3K36me3 (Kraushaar et al., 2013; Lin et al., 2013), but the correlation between H3.3 and ncH3K4me3 is unclear and whether the replacement of H3.3 is responsible for the removal of ncH3K4me3 deserves further investigation.

The canonical form of H3K4me3 in the promoter region is established at the 2-cell stage. Interestingly, the width of H3K4me3 peaks is highly dynamic during mouse pre-implantation embryonic development and is positively correlated with gene expression level (Liu et al., 2016b). The dynamicity of the extent of H3K4me3 marks may provide a novel mechanism of epigenetic regulation in early cleavage embryos when most repressive makers are removed from the promoter region (Liu et al., 2016b). Broad H3K4me3 domains (> 5 kb) in the promoters are much more abundant in early embryos than in MII oocytes or derived cell lines. Lack of KDM5B can extend the width of promoter H3K4me3 domains and disrupt precise lineage differentiation, which indicates that broad promoter H3K4me3 marks may help maintain the transcription of key cell-type-specific factors in a steady state (Liu et al., 2016b). The mechanism of H3K4me3 reprogramming remains unknown, but a recent study showed that the establishment of broad H3K4me3 domains may be catalyzed by KMT2B, a deficiency of which results in slower ovulation and embryonic arrest (Andreu-Vieyra et al., 2010). However, which transcription factors are involved in this precisely controlled process requires further investigation.

#### Canonical pattern of H3K4me3 in GV oocytes and species-specific dynamics of H3K4me3 in humans

**Figure 2:**
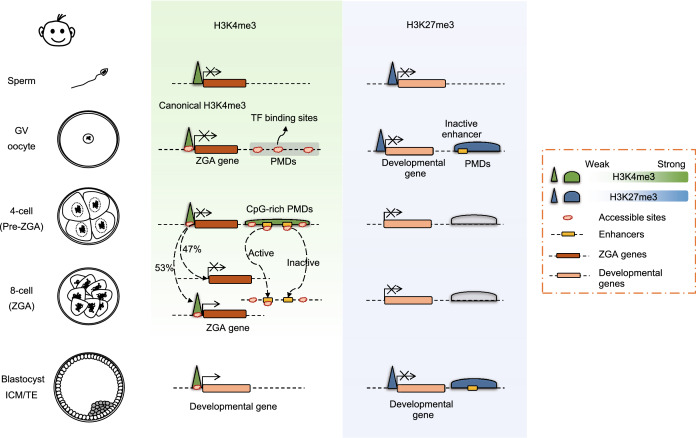
**Dynamic histone modifications and chromatin accessibility during human pre-implantation embryogenesis.** H3K4me3: H3K4me3 shows sharp peaks at promoters in human GV oocytes. During early human embryogenesis, wider promoter H3K4me3 marks are easily observable at the 4-cell stage (pre-ZGA), and 53% of these marks remain at the 8-cell stage (peri-ZGA) and are preferentially activated. The remainder of these sites (~ 47%), where H3K4me3 is lost, are in the promoters of genes related to development and differentiation, which remain inactive during ZGA. Weaker (compared to promoter H3K4me3) but widespread distal H3K4me3 marks are observed in pre-ZGA embryos, which indicates the *de novo* deposition of H3K4me3, and are decreased at the 8-cell stage. Distal H3K4me3 is deposited in CpG-rich and hypomethylated regions. Most of the distal H3K4me3 marks overlap with *cis*-regulatory elements and are highly chromatin accessible at the 4-cell stage. H3K27me3: H3K27me3 in human GV oocytes is deposited in the promoters of developmental genes and partially methylated domains. Human embryos at the ZGA stage (8-cell stage) show almost no H3K27me3 signals, indicating the global erasure of H3K27me3 in the maternal genome. The inaccessible *cis*-regulatory elements located in distal chromatin regions near developmental genes are correlated with H3K27me3 in the human ICM. Chromatin accessibility: Widespread accessible chromatin regions also highly overlap with *cis*-regulatory elements and transposable elements in human early embryos. High chromatin accessibility exists in distal regions enriched in transcription factor binding sites, overlapping with hypomethylated DNA regions in oocytes. Distal accessible chromatin at the 4-cell stage is also enriched for distal H3K4me3. These distal regions become inaccessible after the 8-cell stage

The CUT&RUN (cleavage under targets and release using nuclease) method (Skene and Henikoff, 2017) reduces the requirement for materials for the genome-wide analysis of histone modifications. Thus, the reprogramming of key histone modifications in human pre-implantation embryo development is finally accessible to profiling (Fig. 2). Unexpectedly, H3K4me3 was found to show sharp peaks at promoters in human GV and MI oocytes (Xia et al., 2019), unlike the abundant broad ncH3K4me3 domains distributed in distal partially methylated regions in mouse mature oocytes. These differences indicate a different epigenetic mechanism of genome silencing in human oocytes that is regulated by ncH3K4me3 in mice. Notably, weaker (compared to promoter H3K4me3) but widespread distal H3K4me3 marks have been discovered in pre-ZGA embryos, which indicates the de novo deposition of H3K4me3 and characterizes a primed epigenetic state for subsequent transcriptional regulation (Xia et al., 2019). The function of these distal H3K4me3 marks remains unknown, and whether their function is similar to that of ncH3K4me3 in mice before ZGA is worthy of elucidation. The profiling of such major reprogramming of both promoter and distal H3K4me3 marks will pave the way for future investigations of parental-to-zygotic transition in human pre-implantation embryos.

### H3K27me3

#### Large-scale loss of promoter H3K27me3 marks after fertilization and pervasive ncH3K27me3 marks

H3K27me3 is a polycomb-based chromatin signature associated with gene repression (Bernstein et al., 2006; Simon and Kingston, 2009; Di Croce and Helin, 2013). H3K27me3 has been proved to be intergenerationally inherited from the maternal genome during early embryogenesis, regulating the activation of enhancers and lineage-specific genes (Inoue et al., 2017a; Zenk et al., 2017). During mouse early embryo development, H3K27me3 in promoter regions of both maternal and paternal alleles exhibits a widespread loss as early as in PN5 zygotes, followed by rapid dynamics during the transition from the morula to inner cell mass (ICM) and trophectoderm (TE) stages (Santos et al., 2005; Liu et al., 2016b; Zheng et al., 2016; Inoue et al., 2017a) (Fig. 1). This rapid loss is accomplished by the global erasure of H3K27me3 in the paternal genome and the selective depletion of promoter H3K27me3 in the maternal genome. In addition, dual-omics analysis indicate a negative correlation between H3K27me3 and DNA methylation in MII oocytes (Zheng et al., 2016) and some genomic regions in post implantation embryos (Yang et al., 2018b), while the anti-correlation is indistinct in cultured cells like ESCs and neural stem cells (NSCs) (Bartke et al., 2010; Wu et al., 2010; Brinkman et al., 2012), as well as mouse pre-implantation embryos, in which H3K27me3 is negatively correlated with H3K4me3 (Liu et al., 2016b; Zheng et al., 2016). On the other hand, H3K27me3 also appears in non-promoter regions, where it is highly pervasive and dramatically promiscuous. This supports the hypothesis that polycomb complexes constantly scan the whole genome for H3K27me3 deposition to compensate for the transcription repression function, which can also be realized by DNA methylation and H3K9me3. However, the functions and molecular mechanisms of such non-canonical H3K27me3 patterns remain to be verified.

In SCNT embryos, strong H3K27me3 signals are observed in all pseudopronuclei at the 1-cell stage (Zhang et al., 2009; Xie et al., 2016), which are highly distinct from the asymmetric H3K27me3 marks in parental genomes in normally fertilized embryos. In addition, ZGA failure in SCNT embryos at the 2-cell stage seems to be compatible with the defect of H3K27me3 erasure inherited from donor somatic cells, suggesting that H3K27me3 represents an epigenetic barrier to epigenome reprogramming (Zhang et al., 2009; Matoba et al., 2018). But simply eliminating H3K27me3 signals does little to promote the SCNT efficiency. A recent study shows that the overexpression of *Kdm6a* (an H3K27me3 demethylase) facilitates the expression of ZGA-related genes in SCNT embryos but results in no improvement of the birth rate or ntESC establishment efficiency. Conversely, knockdown of *Kdm6b* (another H3K27me3 demethylase) promotes both ZGA and SCNT efficiency (Yang et al., 2018a). H3K27me3-dependent imprinting genes are aberrant in SCNT embryos (Okae et al., 2014b; Inoue et al., 2017a) and may be amended by knockdown of *Kdm6b*. *Kdm6b* reduction also disrupts the ectopic expression of *Xist*, in which H3K27me3 serves as an imprinting mark and is correlated with abnormality of X chromosome inactivation in SCNT embryos (Inoue et al., 2010; Matoba et al., 2011; Inoue et al., 2017b; Matoba et al., 2018; Yang et al., 2018a).

Maternal H3K27me3-mediated imprinting has been discovered in early mouse embryos (Inoue et al., 2017a), as it can mediate silencing of DNA hypomethylated promoters, achieved by polycomb recruitment (Deaton and Bird, 2011). The H3K27me3 imprinting is probably established during oogenesis and maintained in pre-implantation embryos, which will be diluted in ICM and most of the H3K27me3-mediate imprinting is lost in the epiblast (EPI) of E6.5 embryos (Inoue et al., 2017a). Recent studies reveal that oocyte-derived H3K27me3-mediated imprinting will switch to being DNA methylation-dependent in extra-embryonic cells after implantation, in which both maternal EED and zygotic DNMT3A/B may take part (Smith et al., 2017; Inoue et al., 2018; Chen et al., 2019b), reflecting the complementary roles of H3K27me3 and DNA methylation in controlling imprinting. Recently, the complete loss of H3K27me3-dependent imprinting patterns in maternal allele-specific genes has been found in SCNT blastocysts, probably due to the absence of H3K27me3 marks at these loci in donor somatic cells (Okae et al., 2014b; Matoba et al., 2018). These epigenetic abnormalities of pre-implantation SCNT embryos are highly correlated with subsequent placenta overgrowth and embryonic lethality (Miri et al., 2013; Okae et al., 2014b; Inoue et al., 2017b). Intriguingly, the upregulation of clustered miRNAs of *Sfmbt2*, one of the H3K27me3-dependent imprinted genes (Matoba et al., 2018; Chen et al., 2020), has recently been identified as the major cause of placental hyperplasia in SCNT mice (Inoue et al., 2020). These observations highlight the crucial function of H3K27me3-imprinted genes in both pre-implantation and postimplantation embryos, although the detailed molecular mechanisms and consequences of intergenerational epigenetic inheritance remain elusive.

#### Global erasure of H3K27me3 at human ZGA

H3K27me3 in human GV oocytes is deposited in promoters of developmental genes and partially methylated domains, differing from the pattern in mouse oocytes (Xia et al., 2019). During human pre-implantation embryo development, the resetting of H3K27me3 is also different from that in mice. Human embryos at ZGA (8-cell stage) show almost no H3K27me3 signal, indicating the global erasure of H3K27me3 on both parental genomes (Xia et al., 2019) (Fig. 2). The absence of the core components of polycomb repression complex 2 (PRC2) in human embryos may be correlated with the loss of H3K27me3 (Saha et al., 2013). On the other hand, the global loss of H3K27me3 in human embryos predicts the absence of imprinting regulation, such as X chromosome inactivation (XCI), which is one of the critical events during early mouse embryogenesis (Petropoulos et al., 2016; Inoue et al., 2017b). Moreover, the H3K27me3-mediated imprinted genes identified in mouse early embryos, which have human orthologs, seems to experience *de novo* deposition of H3K27me3 (Xia et al., 2019). Further study is required to verify whether H3K27me3-controlled imprinting exist in human early embryos. Intriguingly, an analysis revealed asymmetric H3K27me3 patterning between ICM- and TE-specific genes in human pre-implantation embryos. Considering that ICM samples are a mixture of EPI and PE (primitive endoderm) cells, further studies are urgently needed to determine whether preferential H3K27me3 deposition also exists between EPI and PE cells (Xia et al., 2019).

#### Bivalent H3K4me3 and H3K27me3

Bivalent domains are considered to provide developmental regulators for later transcriptional activation upon differentiation (Vastenhouw and Schier, 2012). Although an H3K4me3/H3K27me3 bivalent state has been observed in lineage control genes in ESCs (Bernstein et al., 2006), zebrafish blastomeres (Vastenhouw et al., 2010), and primordial germ cells (PGCs) (Sachs et al., 2013), whether the associated regulatory characteristics differ remains unclear. It has been shown that the number of bivalent peaks is much lower in mouse pre-implantation embryos than in ESCs (Liu et al., 2016b), which indicates that bivalency may be more important in a stable cell line than in a transient stage. Bivalency is absent in developmental genes and is not established until the blastocyst stage, when lineage differentiation begins. Notably, most bivalent ICM and TE genes can be inherited by ESCs and TSCs and exhibit lower levels of expression (Liu et al., 2016b; Zheng et al., 2016). It is worth mentioning that EZH2 and SUZ12, the core components of PRC2, which are responsible for H3K27me3 deposition, target most of the inherited bivalent genes (Margueron and Reinberg, 2011; Liu et al., 2016b). This finding may help us understand the regulatory pattern of PRC2-mediated H3K27me3 upon the exit from totipotency to differentiation in early embryo development.

There is an absence of H3K4me3 and H3K27me3 bivalency in developmental genes upon implantation (Zheng et al., 2016). Intriguingly, in the EPI on embryonic day 6.5 (E6.5), stronger bivalency, defined as “super bivalency”, is found in the promoters of developmental genes, which is also evident in the E7.5 ectoderm but is much weaker in the E6.5 visceral endoderm (VE), mESCs and somatic lineages (Xiang et al., 2020). This raises the question of the function of this transient super bivalent state. Evidence shows that “super bivalency” is correlated with lineage-specific gene activation at later stages, such as cortex or heart differentiation. In addition, these super bivalent genes in the E6.5 EPI exhibit a unique higher-order chromatin organization (Xiang et al., 2020). These findings raise the possibility that strong bivalency in the primed EPI can help key developmental genes to maintain a unique spatial distribution and remain in a poised state for activation. KMT2B (MLL2) is known to be a methyltransferase for ncH3K4me3 in mouse oocytes (Hanna et al., 2018) and is responsible for the deposition of H3K4me3 in bivalent regions. In *Kmt2b* knockout embryos, H3K4me3 is globally downregulated at bivalent promoters, which is associated with aberrant developmental gene activation. Unexpectedly, bivalency is partially restored in some fraction of developmental gene promoters in the E8.5 head, indicating that *Kmt2b* plays a critical role in the super bivalency of the E6.5 EPI, while compensation mechanisms are involved in later embryonic development (Xiang et al., 2020). Furthermore, DNA hypomethylation is also suggested to participate in bivalency maintenance, in that *Tet1/2* double knockout (DKO) leads to increased DNA methylation levels, followed by a significant H3K4me3 decrease in bivalent developmental genes (Xiang et al., 2020). Taken together, these results reveal a unique chromatin state transition that specifically appears during development from pre-implantation embryos to cell fate-determined lineages.

### H3K9me3

#### H3K9me3 is a barrier to cell fate transition

H3K9me3-dependent heterochromatin is regarded as a barrier to cell fate changes, as it occludes the DNA from transcription factor binding (Tachibana et al., 2002; Burton and Torres-Padilla, 2010; Soufi et al., 2012; Becker et al., 2016). The principles and mechanisms of H3K9me3-dependent heterochromatin formation and function have recently been well reviewed elsewhere (Allshire and Madhani, 2018).

Early study has discovered gradual but incomplete demethylation patterns of H3K9me2 and H3K9me3 in SCNT embryos, in contrast to the asymmetric ones present in the parental genomes of fertilized embryos (Wang et al., 2007). The aberrant H3K9me3 reprogramming is deemed to directly causes ZGA failure, especially in SCNT embryos (Schultz, 2002; Matoba et al., 2014). At the 2-cell stage, reprogramming-resistant regions (RRRs) marked by H3K9me3 are defined, which are regions inherited from donor cells that fail to be successfully reprogrammed upon embryonic development (Matoba et al., 2014). *Kdm4d* (an H3K9 demethylase) overexpression in embryos and *Suv39h1*/*h2* (H3K9me3 methyltransferases) knockdown in donor cells can rescue the transcription of ZGA-related genes impeded by H3K9me3, which dramatically improves the developmental rate of blastocysts. Notably, subsequent experiments using an embryo biopsy system and single-cell transcriptome sequencing identified *Kdm4b*, another H3K9me3 demethylase, as a key factor in the 2-cell arrest of SCNT embryos (Matoba et al., 2014; Liu et al., 2016a). Both studies emphasize the critical role of H3K9me3 reprogramming during early embryogenesis. The analysis of H3K9me3 in donor cells and 2-cell embryos showed a reduction in resistant H3K9me3 signals in *Kdm4d*-overexpressing SCNT embryos (Matoba et al., 2014). Moreover, overexpression of KDM4A also drastically increased the blastocyst rate of human SCNT and improves the derivation of human ntESC, with the similar mechanism as in mice (Chung et al., 2015). Our recent study also shows that H3K9me3 in donor cells also prevent the removal of topologically associated domains (TADs) during SCNT (Chen et al., 2020). These findings suggest that some H3K9me3, mainly associated with heterochromatin, is resistant to the cell conversion methods and presents as a barrier to reprogramming to pluripotency that impairs both the efficiency of reprogramming and the quality of embryogenesis.

On the other hand, H3K9me3 modification regulates the expression of repeats elements and some protein coding genes in mouse pre-implantation embryos (Hatanaka et al., 2015; Wang et al., 2018a). *Kdm4b* overexpression can improve cloning efficiency, but injection of high levels of *Kdm4b* mRNA have been proved to interrupt TE differentiation and an optimized *Kdm4b* mRNA injection dose can further increase the implantation and birth rates of cloned mice (Liu et al., 2016a). These indicate the level of H3K9me3 modification need to be well balanced during reprogramming and embryo development.

#### H3K9me3 regulates the proper expression of LTRs in early mouse embryos

Upon fertilization, a large fraction of repeat-rich sequences, including LTR retrotransposons, undergo dramatic demethylation and become accessible for highly active transcription, which has been shown to be critical for ZGA (Peaston et al., 2004; Wang and Dey, 2006; Zhang et al., 2019). LTRs must be properly regulated because they pose a risk to genome integrity through their potential for illicit recombination and self-duplication. Since major DNA demethylation occurs upon fertilization, the regulation of the transcription of LTRs requires a switch from DNA methylation to other types of epigenetic modifications, including repressive histone modifications, H3K9me3 and H3K27me3 (Wang et al., 2014; Becker et al., 2016). Previous research has shown that CAF-1 is responsible for the deposition of repressive histone marks, including H4K20me3 and H3K9me3, in retrotransposon regions and results in the silencing of retrotransposons in the mouse morula (Hatanaka et al., 2015; Ishiuchi et al., 2015), which provides a possible mechanism of retrotransposon regulation in early embryos. Recently, a high-resolution map for investigating the reprogramming of H3K9me3-dependent heterochromatin in mouse pre-implantation embryos was profiled (Wang et al., 2018a). As expected, it was found that the H3K9me3 peaks fall mainly within LTRs in early embryos and the number of H3K9me3-marked LTRs gradually increases, which remains high during pre-implantation development (Wang et al., 2018a). In contrast, promoter H3K9me3 marks are erased upon fertilization and will be established postimplantation, indicating the involvement of different regulatory mechanisms from those of H3K9me3 in LTRs (Wang et al., 2018a) (Fig. 1). The distribution characteristics of H3K9me3 echo its function in LTR regulation. Further analysis showed that increased H3K9me3 levels are correlated with the silencing of LTRs in the corresponding regions. The proper decoration of H3K9me3 by the CHAF1A complex in LTRs may be critical for blastocyst formation, cell fate decisions and the totipotency-pluripotency transition (Wang et al., 2018a). These results shed light on the molecular mechanisms of LTR silencing by H3K9me3-dependent heterochromatin. Further studies are required to reveal the molecular mechanisms of H3K9me3-dependent chromatin organization for transposable element (TE) regulation in mammalian embryos.

At the postimplantation stage, H3K9me3 marks are re-established in promoter regions. The expression level of lineage-specific genes is repressed owing to the formation of H3K9me3-decorated chromatin in their promoter regions (Wang et al., 2018a). Motif enrichment analysis indicated that *Pou5f1*, *Sox12*, *Sox11*, *Lhx1*, *Zfp105* and *Foxa2* are potential transcription factors contributing to the formation of epiblast-specific H3K9me3, while *Zbed6*, *Elf4*, *Glis2*, *Creb3l2* and *Ascl2* are involved in extraembryonic-specific H3K9me3 formation. Whether these transcription factors all play roles in chromatin organization during embryogenesis remains to be verified. Excitingly, endoderm-specific triple-knockout mutant (TKO) mice for all three histone H3K9 methyltransferases (SETDB1 and SUV39H1/H2) show a three-fold reduction in body weight and are much smaller in size compared to the control mice (Nicetto et al., 2019). In addition, the livers of the TKO mice show remarkable derepression of nonhepatic genes and fail to express mature hepatocyte genes (Nicetto et al., 2019). This highlights the significance of the precise regulation of H3K9me3 deposition at lineage-specific genes during mammalian embryo development.

### OTHER HISTONE MODIFICATIONS

#### H3K36me3

H3K36me3, catalyzed by SETD2, is associated with transcriptionally active chromatin (Edmunds et al., 2008). SETD2 can mediate RNA polymerase II interaction and couple H3K36me3 with transcript elongation (Kizer et al., 2005). Unlike H3K4me3, H3K36me3 positively correlates with DNA methylation at gene bodies by recruiting DNMT3A/B (Baubec et al., 2015), which is conserved in most mammalian cells (Hawkins et al., 2010). SETD2 depleted oocytes exhibit huge loss of H3K36me3 and leads to H3K4me3 and H3K27me3 invasions into the former H3K36me3 regions (Xu et al., 2019). In addition, oocytes lacking SETD2 results in an aberrant DNA methylome which includes loss of maternal imprints and aberrant H3K4me3 deposition instead of DNA methylation, especially at ICRs (Xu et al., 2019). Furthermore, SETD2 scarcity results in oocyte maturation defects and embryonic lethality (Xu et al., 2019). These observations emphasize the essential role of H3K36me3 in establishing and safeguarding the maternal DNA methylome during mouse oogenesis and early embryo development.

#### H3R26me2

Mouse ESCs and blastomeres in early embryos with increased H3R26me2, an activating mark, show higher expression of a subset of pluripotency genes, is highly correlated with cell fate decision and pluripotency (Torres-Padilla et al., 2007; Wu et al., 2009; Goolam et al., 2016; White et al., 2016). Overexpression of coactivator-associated-protein-arginine-methyltransferase 1 (CARM1) in mouse ESCs and embryos elevates expression of key pluripotent genes, like *Oct4*/*Pou5f1* (Wu et al., 2009; Goolam et al., 2016), *Nanog* (Torres-Padilla et al., 2007; Wu et al., 2009) and *Sox2* (Goolam et al., 2016; White et al., 2016), of which promoters display detectable levels of H3R17/26 methylation. Differentially expressed H3R26me2 between 4-cell blastomeres are reported to be mediated by the heterogeneous activity of CARM1, with CARM1-activated blastomeres are prone to develop into ICM rather than TE (Torres-Padilla et al., 2007; Parfitt and Zernicka-Goetz, 2010; Shi et al., 2015). Intriguingly, the earliest cell fate decision in mouse early embryos has recently been advanced to emerge as early as late 2-cell stage, when a long noncoding RNA, LincGET, is transiently and asymmetrically expressed in the nucleus from 2-cell to 4-cell stage (Wang et al., 2018b). Notably, LincGET physically binds to CARM1 (Wang et al., 2018b), promoting CARM1 to accumulate in nuclear granules that requires paraspeckle component NEAT1 and its partner P54NRB (Hupalowska et al., 2018). This further gives rise to the increasing of H3R26me2 level, activating ICM-specific gene expression, upregulating transposons, and increasing global chromatin accessibility (Wang et al., 2018b). The mechanisms of H3R26me2 deposition and its potential effect on nuclear organization and lineage allocation during early embryo development require further investigations.

## TRANSPOSABLE ELEMENTS

Transposable elements are repetitive DNA sequences that account for approximately half of the mammalian genome and have been considered deleterious to cells and a cause of cancer or apoptosis (Burns, 2017; Malki et al., 2019). They are silenced to prevent cells from experiencing promiscuous gene activation and potential mutations (Babaian and Mager, 2016; Burns, 2017) resulting from their transposable nature. Most of these elements are maintained in a silenced state by DNA hypermethylation or repressive histone modifications inducing H3K9me2/3 marks (Karimi et al., 2011; Leung et al., 2014). Transposable elements are highly expressed during mouse embryonic genome activation at the 2-cell stage (Evsikov et al., 2004). The proper regulation of retrotransposon expression is critical for the sequential reprogramming of the embryonic genome (Peaston et al., 2004).

### MERVL

ERVs represent almost 10% of the mouse and human genome (Stocking and Kozak, 2008). MERVL is a member of the ERV3 family member that is expressed in both 2-cell-like ESCs and cleavage-stage embryos, where it drives the expression of many transcripts specific to ZGA and totipotency (Kigami et al., 2003; Svoboda et al., 2004; Macfarlan et al., 2012; Wu et al., 2016). The transcription of MERVL is under stringent surveillance to ensure stage-specific regulation during pre-implantation embryo development.

DUX plays critical roles in converting ESCs into a 2C-like state (Hendrickson et al., 2017) by controlling MERVL through the Dux-miR-344-Zmym2/Lsd1 axis (Yang et al., 2020). A previous study suggested that *Dux* is a ZGA inducer, as it is transiently expressed at the early 2-cell stage and robustly activates ZGA-related genes (De Iaco et al., 2017; Whiddon et al., 2017). Developmental pluripotency-associated 2 (DPPA2) and DPPA4 have been proved to drive the expression of *Dux* by directly bind to its promoter and gene body in 2C-like ESCs (Eckersley-Maslin et al., 2019). However, ~ 20% of embryos with zygotic depletion of DUX is able to reach a later embryonic stage without defective ZGA. Recently, Zhang and our group demonstrated that *Dux* is indeed important but is not a prerequisite for *in vivo* early embryo development (Chen and Zhang, 2019; Guo et al., 2019). Indeed, *Dux* deletion delays ZGA and decreases the developmental potential of embryos, but *Dux*-KO mice can survive to adulthood. Furthermore, the prolonged expression of *Dux* leads to developmental arrest and embryo death, which emphasizes the importance of proper silencing and degradation processes (Guo et al., 2019). Despite the absence of *Dux*, MERVL is highly expressed at the mid- and late 2-cell stages, which indicates the existence of compensation mechanisms that are probably regulated by other transcription factors or chromatin remodelers (Guo et al., 2019).

Distinct from the transient but bursting existence of *Dux* at the early 2-cell stage (initiated as early as the zygotic stage (Macfarlan et al., 2012; Deng et al., 2014; Abe et al., 2018)), *Zscan4c*, a transcription factor with zinc finger domains, is expressed at the 2-cell/4-cell stages, which is compatible with the existence of MERVL transcripts. A recent study in mESCs indicated that *Zscan4c* acts as an activator of MERVL and genes playing roles in 2-cell/4-cell embryo (Zhang et al., 2019). *Zscan4c* activates MERVL via direct binding to MT2 (the LTR of MERVL) loci and activates 2-cell/4-cell embryo genes by regulating the enhancer activity of MT2, associated with the increased deposition of H3K4me1, H3K27ac, and H3K14ac (Zhang et al., 2019). In contrast to *Dux*, *Zscan4c* is suggested to reinforce the activation of the 2C-like transcriptional network, rather than drive it (Eckersley-Maslin et al., 2019).

### LINE1

The long interspersed element 1 (LINE1) retroelements are the most abundant class of retroelements in mammals (Richardson et al., 2015). LINE1 is highly expressed during mouse pre-implantation embryo development (Fadloun et al., 2013), which implies its critical roles in gene regulatory networks (Bourque, 2009). Upon fertilization, LINE1 is actively transcribed and peaks at the 2-cell stage. By contrast, the retrotransposition rate of LINE1 is rather low, giving rise to the hypothesis that LINE1 functions in a retrotransposition-independent manner, which has recently been verified (Jachowicz et al., 2017). Chromatin-associated LINE1 is found in mESCs and interacts with a nucleolin-KAP1/TRIM28 complex (Jachowicz et al., 2017; Percharde et al., 2018). Notably, nucleolin is essential for rRNA synthesis and processing (Ginisty et al., 1998) and has been identified as a repressor of *Dux* and the 2C program in ESCs (Gabellini et al., 2002). PRC1.6 and corepressor tripartite motif-containing protein 28 (TRIM28/KAP1) have been shown to directly bind and repress *Dux* in mESCs (Cossec et al., 2018; Percharde et al., 2018). The reduced abundance of LINE1 RNA affects the interaction between *Dux* and nucleolin in peri-nucleolar heterochromatin, which probably facilitates entry into the 2C state (Percharde et al., 2018). Whether this is consistent in early embryos deserves further *in vivo* exploration. Notably, LINE1 is essential for maintaining an open chromatin state in early embryogenesis (Jachowicz et al., 2017). Prematurely decreased chromatin accessibility occurs if LINE1 is repressed. In contrast, the prevention of chromatin condensation accompanies artificially prolonged LINE1 transcription (Percharde et al., 2018). Taken together, these findings indicate that LINE1 is essential for *Dux* silencing, the synthesis of rRNA, exit from the 2-cell stage and chromatin remodeling over accessible regions during pre-implantation embryo development. The proper regulation and degradation of LINE1 transcripts are critical for normal oocyte maturation and embryogenesis. Our group recently demonstrated a new posttranscriptional regulation mechanism of LINE1 mediated by ZCCHC8, a central factor in the nuclear exosome targeting (NEXT) complex(Wu et al., 2019). *Zcchc8*-depleted ESCs exhibit defects in proliferation, pluripotency maintenance, and differentiation. Intriguingly, the maternal loss of ZCCHC8 in mouse oocytes and early embryos indicates sustained abundant LINE1 RNA, accompanied by higher chromatin accessibility (Wu et al., 2019). ZCCHC8 has been reported to show a functional correlation with RNA export and translation (Lubas et al., 2011; Roundtree et al., 2017; Kasowitz et al., 2018; Mure et al., 2018); thus, the further investigation of RNA modifications may shed light on the underlying regulatory mechanisms.

## CHROMATIN REMODELING

### Chromatin accessibility

During mammalian pre-implantation development, major chromatin reorganization is critical for epigenetic reprogramming to convert terminally differentiated gametes into a totipotent state (Burton and Torres-Padilla, 2014). When global transcription takes place, open chromatin is newly established, while epigenetic modifications can be partially inherited from maternal genomes (Tsompana and Buck, 2014; Wu et al., 2016). The molecular mechanism of how cell memory of open chromatin can be erased and re-established during early embryo development remains unclear. Recent work based on low-input DNase I sequencing (liDNase-seq) suggests that DNase I-hypersensitive sites (DHSs) are progressively established and show a major increase in 8-cell embryos (Jin et al., 2015; Lu et al., 2016). Another study using improved ATAC-seq (an assay for transposase-accessible chromatin using sequencing) revealed the landscape of chromatin accessibility dynamics in early mouse embryo development (Buenrostro et al., 2015; Cusanovich et al., 2015; Wu et al., 2016) (Fig. 1). In contrast to the asymmetric reprogramming patterns of DNA methylation and histone modifications in the parental genomes after fertilization, chromatin accessibility seems to be more synchronized, except for a few instances of allele-specific open chromatin and transcription (Tsompana and Buck, 2014; Wu et al., 2016). Notably, open chromatin exists around both the promoters and transcription end sites of actively transcribed genes at the 2-cell stage, which is distinguished from the map of cis-regulatory sequences in other mouse tissues and cell types (Shen et al., 2012). Additionally, the transient and active transcription of transposable elements is probably associated with increased chromatin accessibility at the 2-cell stage of early embryos (Peaston et al., 2004). In addition, by combining the analysis of transcriptome and chromatin accessibility, NR5A2, which promotes the expression of *Pou5f1* and *Nanog*, has been shown to participate in the regulatory network involved in the lineage specification of ICM and TE, and this regulation may occur as early as the 8-cell stage (Lu et al., 2016; Wu et al., 2016). These findings provide hints about the regulatory circuity during pre-implantation embryogenesis, while multiomics analyses are necessary to complement the panorama of epigenome remodeling.

During SCNT embryo development, chromatin accessibility undergoes major reprogramming and is mostly completed before the first embryonic cleavage (Djekidel et al., 2018). Additionally, this reprogramming appears to be DNA replication independent, indicating the importance of maternal factors involved in chromatin remodeling (Djekidel et al., 2018). The search for the maternal factors responsible for the chromatin state transition is an important research topic that deserves increasing attention.

In human embryogenesis, widely spread accessible chromatin regions are readily detected in 2-cell embryos, before ZGA occurs around the 4-cell to 8-cell stage (Lee et al., 2014; Wu et al., 2018). These regions show a preference for CpG-rich promoters and are correlated with gene activation. High chromatin accessibility also exists in distal regions, which are enriched in transcription factor binding sites and overlap with hypomethylated DNA regions in oocytes. Distal accessible chromatin at the 4-cell stage is also enriched in distal H3K4me3 (Xia et al., 2019) (Fig. 2). Putative transcription factors such as CTCF, KLF, SOX2, POU5F1, GATA and TEAD are well conserved in both mice and humans according to transcriptome or chromatin accessibility analysis (Xia et al., 2019). However, the low-input ATAC-seq method cannot avoid aneuploid human embryos, and the heterogeneity of individuals is much more extensive because of their complex genetic backgrounds. It is worth mentioning that the single-cell chromatin overall omic-scale landscape sequencing (scCOOL-seq) technique, which enables the simultaneous analysis of the chromatin state, nucleosome positioning, DNA methylation, and copy number variation in the same individual cell, has been developed (Li et al., 2018a). The results obtained using this approach indicated that the most dramatic chromosome remodeling in human embryos occurs between the 4- and 8-cell stages (Li et al., 2018a). Studies of DNA methylation and chromatin accessibility in individual cells from the same embryo demonstrate cell-to-cell variance and the unsynchronized reprogramming of the DNA methylome and chromatin organization in different genomic regions. Collectively, these data pave the way for deciphering the mechanism of epigenomic reprogramming in pre-implantation human embryos and indicate potential applications in clinical diagnosis.

### 3D chromatin

The nucleus of eukaryotic interphase cells contains chromatin packaged in a hierarchical structure, which is essential for gene regulatory networks (Fullwood et al., 2009; Atlasi and Stunnenberg, 2017). The 3D chromatin architecture plays critical roles in wide-ranging biological events, including RNA transcription, DNA replication, cell division and meiosis (Gorkin et al., 2014; Bonev and Cavalli, 2016; Beagrie et al., 2017; Hug and Vaquerizas, 2018). The role of the 3D genome organization in development and cell differentiation has been reviewed in detail elsewhere (Zheng and Xie, 2019). Nevertheless, the dynamics of the 3D chromatin structure during mammalian embryogenesis remain elusive, owing to the limited technology available for investigating the 3D genome organization.

#### Substantially distinct 3D structures in gametes and gradually established chromatin organization during embryogenesis

**Figure 3:**
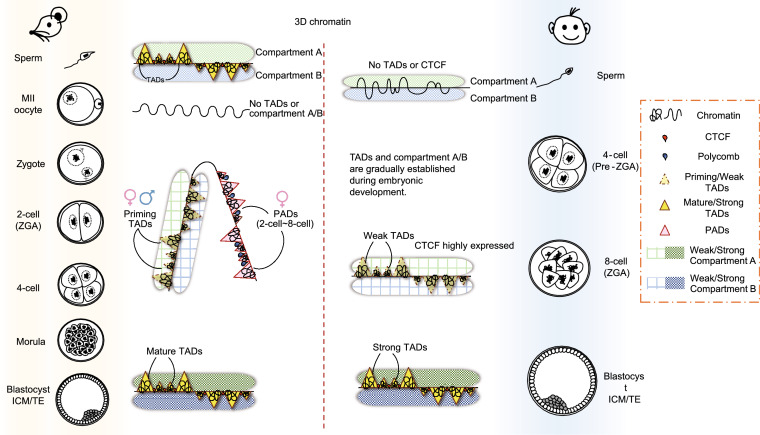
**The higher-order chromatin organization in the gametes and pre-implantation embryos of mouse and human.** Mouse: 3D chromatin: MII oocytes lacks TADs and compartments given its mitotic. PADs and their compartmental interactions appear to emerge only on the maternal allele in early 2-cell embryos, and begin to fade away in the 8-cell embryos. By contrast, sperm present frequent extra-long-range interactions (> 4 Mb) and inter-chromosomal interactions. Upon fertilization, the higher-order structures of both parental chromatins are obscure at the zygotic and ZGA stages but are spatially separated from each other with distinct compartmentalization. The gradual establishment of the parental chromatin organization occurs throughout the development of pre-implantation embryos, with slow consolidation of TADs and the A/B compartments. Such allele separation and compartmentalization are maintained until the 8-cell stage. **Human**: Human sperm lack TADs and expression of the chromatin regulator CTCF. Consistent with findings in mice, TADs and A/B compartmentalization are gradually established during human embryogenesis. CTCF is highly expressed at the ZGA stage of human embryos, which coincides with the time at which TADs are observed

Recent studies utilizing the low-input Hi-C (genome-wide chromosome conformation capture) method revealed the dynamics of 3D genome architecture reprogramming in mouse early embryo development (Du et al., 2017; Ke et al., 2017) (Fig. 3). Notably, MII oocytes lacks TADs and compartments given its mitotic nature, while earlier stages of oocytes show Polycomb-associating domains (PADs), which is marked by H3K27me3 and briefly reappear on the maternal genome upon fertilization (Du et al., 2020). By contrast, sperm present both topologically associating domains (TADs) and A/B compartments (Du et al., 2017). The sperm genome also shows frequent extra-long-range interactions (> 4 Mb) and inter-chromosomal interactions(Battulin et al., 2015). Following fertilization, the higher-order structures of the chromatin of both parents are obscure at the zygotic and ZGA stages but are spatially separated from each other and show distinct compartmentalization. Such allele separation and compartmentalization are maintained until the 8-cell stage and coincide with accumulations of H3K27me3 ( Du et al., 2017; Borsos et al., 2019; Collombet et al., 2020). The gradual establishment of the parental chromatin organization occurs throughout the development of pre-implantation embryos, with slow consolidation of TADs and the A/B compartments. Notably, the re-establishment of chromatin compartments in the maternal genome appears to be much weaker than that in the paternal genome, which is compatible with the distinct 3D nature of the chromatin of oocytes and sperm to some extent (Du et al., 2017). In addition, the lamina-associated domains (LADs) (van Steensel and Belmont, 2017) of both parental genomes experience *de novo* but distinct establishment (Borsos et al., 2019), which precedes consolidation of TADs.

Dramatic higher-order chromatin structure reprogramming indicates the importance of epigenetic reorganization during mammalian early development. However, little is known about the molecular basis of the reprogramming of the 3D chromatin structure. The establishment of TADs depends on DNA replication rather than ZGA, which reveals the function of cell cycle transition in genome structure dynamics (Ke et al., 2017). Furthermore, the reprogramming of other epigenetic modifications is dramatically interrelated with the reorganization of the 3D genome during mouse early embryogenesis. Large-scale DNA demethylation, for instance, appears to preferentially occur in compartment A rather than compartment B, resulting in an increase in the accessibility of chromatin to DNA demethylases (Du et al., 2017; Ke et al., 2017).

Moreover, a recent study by our group revealed spatiotemporally dynamic chromatin reorganization in SCNT embryos (Chen et al., 2020). Both aberrant TADs and compartment A/B organization are observed throughout early SCNT embryo development. The overexpression of *Kdm4b* partially ameliorates abnormal 3D chromatin structures, suggesting that H3K9me3 modification in donor cells is a barrier to chromatin structure reprogramming. This indicates a correlation between the organization of the 3D genome architecture and histone modifications.

#### No TADs or CTCF-dependent 3D chromatin structure in human sperm

The dynamics of the 3D chromatin architecture during human embryogenesis have recently been revealed. Unlike mouse sperm, human sperm lack TADs and the expression of the chromatin regulator CTCF (Chen et al., 2019a) (Fig. 3). Consistent with findings in mice, TADs and A/B compartmentalization are gradually established during human embryogenesis. *CTCF* is highly expressed at the ZGA stage of human embryos, which coincides with the time at which TADs are observed, indicating the critical role of *CTCF* in the establishment of the higher-order chromatin structure during early human embryo development. Intriguingly, the establishment of TADs in human embryos is ZGA dependent, which leads to the hypothesis that the correlation between TAD formation, DNA replication and gene activation differs among species or cell types (Ke et al., 2017; Chen et al., 2019a).

## PERSPECTIVES

During the development of pre-implantation embryos, ZGA occurs predominantly at the two-cell stage in mice and the eight-cell stage in humans, which involves global DNA demethylation, chromatin remodeling, spatial reorganization of the genome and substantial transcriptional changes. Recent discoveries have shed light on the potential regulatory mechanisms that drive subsequent biological events upon fertilization, but many such mechanisms remain to be investigated. Future work is needed to further elucidate the molecular details that underlie the interaction between epigenetic remodeling and the totipotency transition as well as cell fate decisions in early mammalian embryo development.

Epigenetic reprogramming during early embryonic development is an exquisitely controlled process, involving both global re-establishment of most epigenetic marks and locus-specific regulation. In recent years, due partly to single-cell and low-cell-number epigenomic studies, our understanding of the epigenetic reprogramming landscape of pre-implantation development has improved considerably. However, how reprogramming is regulated at different genome loci remains unknown. Different transcription factors must play important roles in determining the locus-specific epigenetic transition pattern. The identification of these factors and the underlying mechanisms will improve our understanding of cell fate transitions and mammalian early development. Cell fate transitions are coordinated by the synergistic action of multiple types of epigenetic remodeling. Multiomics analysis is needed to clarify the fundamental principles underlying totipotency acquisition and cell fate decisions.

The mechanism of epigenetic remodeling seems to vary in different species; for example, TAD establishment depends on DNA replication in mouse embryos, while that in human embryos requires ZGA. The exploration of the epigenetic regulation mechanism of human early embryo development will continue to be an important area of research, the results of which will be beneficial for diagnosing and treating human infertility. It is worth mentioning that human embryos show high individual heterogeneity, including a high proportion of aneuploid embryos, which may to some extent conceal the detailed characteristics of the histone modifications and 3D chromatin structures that exist during human embryogenesis. Notably, remarkable progress has recently been made in mapping the molecular architecture of lineage specification during gastrulation and early organogenesis in mouse embryos (Peng et al., 2019; Pijuan-Sala et al., 2019; Weinreb et al., 2020). Inspiringly, it is now possible to study the development of postimplantation primate embryos through *in vitro* culture (Ma et al., 2019; Niu et al., 2019). This breakthrough may also provide insight into studies on pre-implantation development.

In summary, recent advances in sequencing technologies have given rise to research probing the regulatory network based on epigenome reprogramming during mammalian pre-implantation embryonic development, and further studies are urgently needed to reveal the underlying molecular mechanisms.
